# Regulatory Mechanisms of the Resistance to Common Bacterial Blight Revealed by Transcriptomic Analysis in Common Bean (*Phaseolus vulgaris* L.)

**DOI:** 10.3389/fpls.2021.800535

**Published:** 2022-01-05

**Authors:** Penghui Yang, Yujie Chang, Lanfen Wang, Shumin Wang, Jing Wu

**Affiliations:** Institute of Crop Sciences, Chinese Academy of Agricultural Sciences, Beijing, China

**Keywords:** common bean, common bacterial blight, RNA-seq, plant resistance, WGCNA

## Abstract

Common bean blight (CBB), primarily caused by *Xanthomonas axonopodis* pv. *phaseoli* (*Xap*), is one of the most destructive diseases of common bean (*Phaseolus vulgaris* L.). The tepary bean genotype PI 319443 displays high resistance to *Xap*, and the common bean genotypes HR45 and Bilu display high resistance and susceptibility to *Xap*, respectively. To identify candidate genes related to *Xap* resistance, transcriptomic analysis was performed to compare gene expression levels with *Xap* inoculation at 0, 24, and 48 h post inoculation (hpi) among the three genotypes. A total of 1,146,009,876 high-quality clean reads were obtained. Differentially expressed gene (DEG) analysis showed that 1,688 DEGs responded to pathogen infection in the three genotypes. Weighted gene coexpression network analysis (WGCNA) was also performed to identify three modules highly correlated with *Xap* resistance, in which 334 DEGs were likely involved in *Xap* resistance. By combining differential expression analysis and WGCNA, 139 DEGs were identified as core resistance-responsive genes, including 18 genes encoding resistance (R) proteins, 19 genes belonging to transcription factor families, 63 genes encoding proteins with oxidoreductase activity, and 33 plant hormone signal transduction-related genes, which play important roles in the resistance to pathogen infection. The expression patterns of 20 DEGs were determined by quantitative real-time PCR (qRT-PCR) and confirmed the reliability of the RNA-seq results.

## Introduction

Common bean (*Phaseolus vulgaris* L.), a legume that originated in the Americas, belongs to the Fabaceae family and is one of the most important edible legumes in the world (Schmutz et al., 2014; Brouwer et al., 2016; Rendón-Anaya et al., 2017). It provides valuable sources of protein, vitamins, and minerals that improve its dietary status and nutritional composition for human consumption, directly or indirectly (Kalavacharla et al., 2011). Moreover, studies have shown that common bean is effective in the prevention and control of many diseases, such as diabetes and obesity, due to the presence of nutrients such as thiamin, folic acid and many phenolic compounds (Celleno et al., 2007; Castro-Guerrero et al., 2016; Thompson et al., 2017; Chen et al., 2019). However, the low to moderate levels of resistance to common bacterial blight (CBB) in common bean have resulted in its yield and quality being severely damaged by CBB, a major yield-limiting factor that is widespread globally (Duncan et al., 2011; Viteri and Singh, 2014). Thus, improving horizontal resistance to CBB in common bean is a major goal of breeders worldwide.

CBB is a seed-borne disease caused by *Xanthomonas axonopodis* pv. *phaseoli* (*Xap*) and *Xanthomonas fuscans* subsp. *fuscans* (*Xff*). The pathogenic bacterium *Xff* can be easily identified as it can produce a brown pigment in milk Tween (MT) agar medium (Sheppard et al., 2007). Although both bacterial strains can produce the same disease characteristics and cause similar damage to plants, studies have shown that *Xap* is a major endemic disease in China (Xu et al., 2013). The primary host of the CBB pathogen is *P. vulgaris* L., and it also infects other *Phaseolus* species of the secondary and tertiary gene pools, such as tepary bean (*Phaseolus acutifolis*) and runner bean (*Phaseolus coccineus*) (Singh and Miklas, 2015). Under high humidity and suitable temperature conditions (25–35°C), CBB can cause very serious disease epidemics, with the most serious disease epidemics occurring at 28°C (Yu et al., 2000). It has been reported that this disease can cause a yield reduction of up to 20–60% in susceptible genotypes, and when conditions are favourable for the disease, 80% yield reduction or even total eradication can occur (Harveson and Schwartz, 2007; Viteri and Singh, 2014). Crop rotation and planting of pathogen-free seeds are significant measures used to prevent CBB infection. However, screening resistant germplasm resources, exploring resistance genes and breeding disease resistant varieties are more effective and efficient methods of managing CBB.

Since CBB resistance is a complex trait that is quantitatively inherited, host genetic resistance is the most effective and environmentally sound approach to control CBB. Although the study of CBB resistance has been ongoing in several common bean and tepary bean genotypes for many years, no CBB resistance gene has been identified. To date, at least 30 quantitative trait loci (QTLs) for CBB resistance have been mapped, and these are primarily located on chromosomes 2, 3, 6, 7, 8, and 11 (Jung et al., 1996; Miklas et al., 2003; Liu et al., 2008; Shi et al., 2011; Viteri et al., 2014; Singh and Miklas, 2015; Xie et al., 2017; Simons et al., 2021). However, only three QTLs associated with the linked molecular markers BC420 on chromosome Pv06, SU91 on Pv08 and SAP6 on Pv10 were considered to have major effects and were used for marker-assisted selection (MAS; Fourie et al., 2011). The BC420 and SU91 resistance loci were both derived from the tepary bean resistant genotype PI 319443, and the SAP6 resistance loci, cosegregating with a dominant CBB resistance gene, were derived from the common bean resistant genotype Montana No. 5 (Singh and Miklas, 2015). These three major QTLs explain a significant proportion of the phenotypic variation. Because certain relatives of common bean, such as *P. coccineus* and *P. acutifolius*, have a higher level of resistance, using interspecific crosses is a useful way to breed resistant lines. The breeding lines HR45 and HR67, which have BC420 and SU91 QTLs with pyramided resistance, were derived from XAN159, which was developed from an interspecific cross between ICI Piajo and *P. acutifolius* PI 319443 (Park and Dhanvantari, 1994; Singh and Miklas, 2015). However, the map of CBB resistance loci obtained using nine different biparental populations showed poor colocalisation, which reflected the high complexity of CBB resistance.

In recent years, transcriptome sequencing has become an important approach for studying gene expression analysis, selecting differentially expressed genes (DEGs), functional gene mining, and identifying key components of disease resistance pathways in plants (Stark et al., 2019). Based on the common bean genome and transcriptome sequencing technologies, all transcriptional responses, structure–function relationships and transcriptional regulation of disease-resistant common bean genotypes can be better studied in a holistic manner. Analysing the transcriptomic response of resistance to CBB is a way to enhance our knowledge of the molecular mechanisms underlying CBB resistance and provide important information for developing genetic management techniques for this disease. In previous reports, the transcriptomic response of the BAT93 and JaloEEP558 common bean genotypes was investigated after infection by *Xanthomonas phaseoli* pv. *phaseoli*, and resistance was found to be associated with the salicylic acid pathway and sugar metabolism; moreover, the genes involved were modified (Foucher et al., 2020). In this study, two common bean genotypes, namely, the resistant genotype HR45 and the susceptible genotype Bilu, and one tepary bean genotype (PI 319443) were subjected to Illumina sequencing to analyse DEGs and resistance pathways in resistant and susceptible genotypes in response to infection by *Xap*.

## Materials and Methods

### Plant Materials and Bacterial Strain

The resistant genotypes HR45 and PI 319443 and the susceptible genotype Bilu were used in the present study (Supplementary Table 1). HR45 was derived from the tepary bean genotype PI 319443, and they both contain two major resistance loci (BC420 and SU91) (Singh and Miklas, 2015). Seeds of the three genotypes were sown in plastic pots (7 cm × 7 cm × 9 cm), with seven plants in each pot and three replicates, using a 1:1 mixture of vermiculite and nutritional soil. For the plant growth conditions, the day/night temperature was set at 25°C/22°C, with a 16 h/8 h light/dark cycle. To provide adequate conditions for pathogen infection, the relative humidity was set at 95% on the day before inoculation.

The *Xap* strain XS2, isolated and preserved by our laboratory, is the most widespread and harmful pathogen in China. To obtain fresh bacterial cultures, suspensions were grown for 48 h in nutrient broth at 28°C at 220 rpm. MT medium was prepared according to the International Seed Testing Association (ISTA) method (Sheppard et al., 2007).

### Pathogenicity Assays

Bacterial suspensions were adjusted to 1 × 10^8^ colony-forming units (CFU)/mL in sterile distilled water. Eight days after planting, *Xap* inoculation was carried out when the primary leaves were fully expanded, and the relative humidity and temperature were increased to 95% and 28°C/26°C (light/dark) to provide the appropriate conditions for inoculation. Pathogen inoculation was conducted by injection as follows: a 200 μL pipette tip dipped in bacterial solution was used to pierce the leaf at three points on the right side at a distance of approximately 0.5 cm from the central main vein, allowing the pathogenic bacteria to enter the leaf through the wound to infect the host tissue; the left side of the leaf was inoculated with sterile water as a control. After the completion of inoculation, the sample was incubated at 28°C with 95% humidity. The severity of disease in each inoculated leaf was investigated and recorded with reference to the rating criteria (Zapata, 2006).

### Quantification of *Xap*

The concentrations of the DNA samples from the three genotypes (PI 319443, HR45, and Bilu) and XS2 were determined. The XS2 DNA solutions were subjected to six serial dilutions using sterilised water to obtain concentrations of 50, 5, 5 × 10^–1^, 5 × 10^–2^, 5 × 10^–3^, and 5 × 10^–4^ ng/μL. The DNA samples of the PI 319443, HR45 and Bilu genotypes were used as solution backgrounds. The specific primer QXS2 for quantitative real-time PCR (qRT-PCR) was used to specifically amplify XS2 DNA (Supplementary Table 2). Using the obtained data, DNA standard curves were plotted, and regression equations were calculated using the C_*T*_ value as the vertical coordinate and the logarithmic value of the DNA sample concentration as the horizontal coordinate. Leaves of the three genotypes inoculated by injection were collected at 7 days post inoculation (dpi). The DNA was extracted, and its concentration was adjusted to 50 ng/μL. qRT-PCR was used to calculate the pathogen content based on the regression equation that was obtained.

### Sample Collection and Illumina Sequencing

The leaves were collected from the plants after inoculation with the CBB pathogen, and the sampling periods were 0, 24, and 48 h post inoculation (hpi). Each genotype was examined three times, and a total of 27 samples were taken for transcriptome analysis. The samples were named with K for tepary bean PI 319443, R for HR45, and S for Bilu. The samples at 0 h were named K0-1, K0-2, K0-3, R0-1, R0-2, R0-3, S0-1, S0-2, and S0-3; the samples at 24 h were named K1-1, K1-2, K1-3, R1-1, R1-2, R1-3, S1-1, S1-2, and S1-3; and the samples at 48 h were named K2-1, K2-2, K2-3, R2-1, R2-2, R2-3, S2-1, S2-2, and S2-3. The total RNA from each sample was extracted using the Plant Total RNA Extraction Kit following the manufacturer’s instructions (Tiangen, Beijing, China). The RNA Nano 6000 Assay Kit for the Bioanalyzer 2100 system (Agilent Technologies, CA, United States) was used for accurate determination of the quantity and quality of the total RNA. The cDNA libraries of 27 high-quality RNA samples were constructed using the Illumina RNA-seq Kit and sequenced by the Illumina NovaSeq 6000 platform according to standard protocols, and 150-bp paired-end reads were generated. Clean reads were obtained by removing reads containing adaptors, reads containing poly-N sequences and low-quality reads from the raw data. Then, the Q20, Q30, and GC levels of the clean data were calculated. All downstream analyses were based on clean data with high quality.

The reference genome model annotation files were downloaded from http://plants.ensembl.org/Phaseolus_vulgaris/Info/Index. The index of the reference genome was built using Hisat2 v2.0.5 (Kim et al., 2019). FeatureCounts v1.5.0-p3 was used to determine the number of reads mapped to each gene (Liao et al., 2014). Then, the FPKM of each gene was calculated based on the length of the gene and read count mapped to this gene. FPKM, the expected number of fragments per kilobase of transcript sequence per million base pairs sequenced, considers the effect of sequencing depth and gene length on the read count at the same time and is currently the commonly used method for estimating gene expression levels.

### Differential Expression Analysis and Functional Enrichment

Differential expression analysis was performed using the DESeq2 R package (1.20.0) (Love et al., 2014). DESeq2 provides statistical routines for determining differential expression in digital gene expression data using a model based on the negative binomial distribution. The resulting *p* values were adjusted using Benjamini and Hochberg’s approach for controlling the false discovery rate. Genes with an adjusted *p* value < 0.05 identified by DESeq2 were assigned as being differentially expressed. Prior to differential gene expression analysis, for each sequenced library, the read counts were adjusted by the edgeR program package through one scaling normalised factor. Differential expression analysis of two conditions was performed using the edgeR R package (3.22.5). The *p* values were adjusted using the Benjamini and Hochberg method. A corrected *p* value of 0.05 and absolute fold change of 2 were set as the thresholds for significantly differential expression.

Gene Ontology (GO) enrichment analysis of DEGs was implemented by the clusterProfiler R package, in which gene length bias was corrected. GO terms with corrected *p* values less than 0.05 were considered to be significantly enriched by DEGs. KEGG is a database resource for understanding the high-level functions and utilities of biological systems, such as cells, organisms and ecosystems, from molecular-level information, especially large-scale molecular datasets generated by genome sequencing and other high-throughput experimental technologies^1^. We used the cluster Profiler R package to test the statistical enrichment of DEGs in KEGG pathways (Yu et al., 2012).

### Weighted Gene Co-expression Network Analysis

Weighted gene co-expression network analysis (WGCNA) is a systematic biological method used to describe the gene association modes among different samples. It can be used to identify gene sets that are highly synergistically changed and identify candidate biomarkers or therapeutic targets based on the coherence of gene sets and the correlation between gene sets and phenotypes. The R package WGCNA (version 3.6.1) is a set of functions used to calculate various weighted association analyses, which can be used for network construction, gene screening, gene cluster identification, topological feature calculation, data simulation, and visualisation (Langfelder and Horvath, 2008).

### Validation of RNA-seq Results by qRT-PCR

To validate the RNA-seq results, qRT-PCR assays were performed on 20 selected genes from the 139 identified resistance-related genes (Supplementary Table 2). For each sample, cDNAs were synthesised from the total RNA using the UEIris II RT-PCR System for First-Strand cDNA Synthesis Kit (Biodee, Beijing, China) according to the manufacturer’s recommendations. qRT-PCR-specific primers were designed by Primer Premier 6.0 software, and the specificity and efficiency of each primer pair were checked by qRT-PCR and by melting curve analysis of serial dilutions of pooled cDNAs from the sample set. qRT-PCR was performed on an Applied Biosystems 7500 instrument by using a SYBR Green TransStart Top Green qPCR SuperMix Kit (TransGen Biotech, Beijing, China) with the following cycling conditions: denaturation at 94°C for 30 s; 40 cycles of denaturation at 95°C for 15 s followed by annealing and extension at 60°C for 34 s. The actin gene was used as a control to calculate the relative gene expression level. Each reaction was carried out in a final volume of 20 μL containing 10 μL of SYBR Green Mix, 0.4 μL of each primer for the selected genes, 2 μL of 100 ng/μL cDNA, and 7.2 μL of ddH_2_O. The qRT-PCR experiment was repeated with three biological replicates for each sample, and the results were analysed by the 2^–ΔΔ*Ct*^ method (Schmittgen and Livak, 2008).

## Results

### Pathogenicity Assays

**FIGURE 1:**
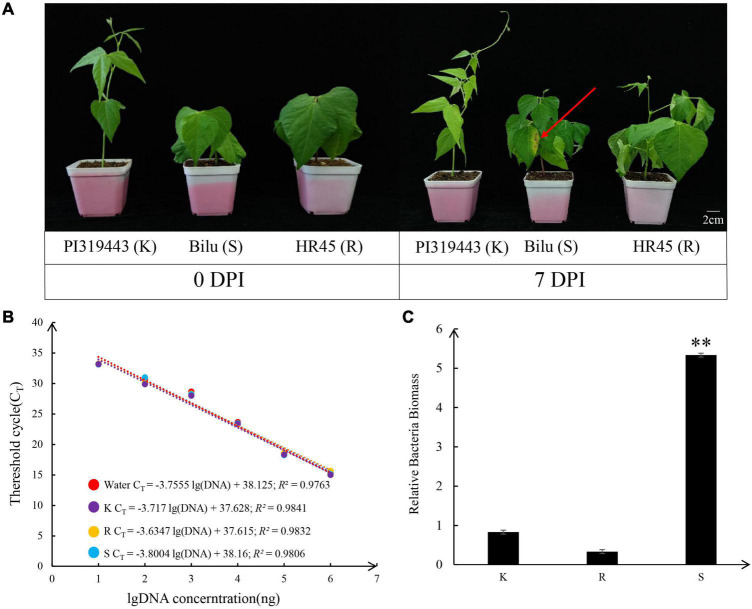
Pathogenicity of strain XS2 on PI 319443, HR45, and Bilu. **(A)** The picture shows the comparison of three genotypes inoculation pathogen for 7 days, and the red arrows indicate the inoculation area on Bilu. **(B)** Standard curve based on different concentrations of *Xanthomonas axonopodis* pv. *phaseoli* strain XS2 DNA with water and three genotypes DNA solution. **(C)** *Xanthomonas axonopodis* pv. *phaseoli* DNA amount in the inoculated leave of three genotypes detected by real time quantitative PCR. Asterisks indicate significantly different at 0.01 probability levels (*p* < 0.01).

The resistance and susceptibility phenotypes of the common bean and tepary bean genotypes HR45, PI 319443 and Bilu were demonstrated with bacterial growth and symptom development after inoculation with *Xap* strain XS2. The results of common bacterial disease resistance identification showed that at 7 dpi, the pathogenicity level in HR45 and PI 319443 was grade 1, and the main feature was that only mechanical damage appeared at the inoculation site without symptoms, while the pathogenicity level in Bilu was grade 6, and the main feature was that transparent water stains appeared at the inoculation site, surrounded by a yellow halo, and expanded to the surrounding area (Figure 1A).

Furthermore, the amount of pathogen colonisation in the inoculated leaves was determined. The R2 values of the regression equations with sterile distilled water, HR45, PI 319443, and Bilu DNA as background were above 0.97, indicating that the results were reliable. The amplification efficiency was close to 1 (100% amplification efficiency), which indicates that the amplification efficiency was close to the ideal state. Colonisation analysis showed that the amount of pathogenic colonisation in Bilu was 6.4 and 15.8 times higher than that in PI 319443 and HR45 (*p* value < 0.05), respectively, while there was no significant difference between the amount of colonisation between PI 319443 and HR45 (Figures 1B,C). The above results indicated that PI 319443 and HR45 were highly resistant to CBB and that Bilu was highly susceptible to CBB. PI 319443 and HR45 are can be widely used as a excellent germplasm resource for resistance breeding and for studying resistance genes and its mechanisms.

### Transcriptome Sequencing and Assembly

Twenty-seven sequencing libraries were generated for total RNA samples of the three genotypes HR45, Bilu, and PI 319443 at three time points (0, 24, 48 hpi). After filtering adaptors and low-quality reads, a total of 1,146,009,876 high-quality clean reads were generated, and each sample yielded approximately 6 Gb of clean reads (Supplementary Table 3). The average GC content was 44.5%. The Q20 and Q30 percentages were more than 97.53 and 93.08%, respectively. Analysis with the common bean G19833 V1.0 reference genome (Schmutz et al., 2014) showed that the total mapping rate of the common bean HR45 and Bilu genotypes ranged from 93.56 to 95.96%, with a mean value of 95.14%, and the unique mapping rate was approximately 90%. In contrast, the total mapping rate of tepary bean PI 319443 was approximately 80%, and the unique mapping rate was approximately 77% (Supplementary Table 4). A total of 28,920 unigenes were obtained after transcriptome assembly.

The expression levels of all these genes were determined based on the expected FPKM values. A total of 22,202 unigenes with an average FPKM > 1 in at least one treatment were considered expressed unigenes and were further analysed (Supplementary Figure 1A). Pearson’s correlation was calculated based on the log_10_(FPKM+1) value, and the correlation coefficients (R^2^ values) of the biological replicates were all above 95%, indicating a high degree of consistency among the replicates (Supplementary Figure 1B and Supplementary Table 5).

### Identification of DEGs

**FIGURE 2:**
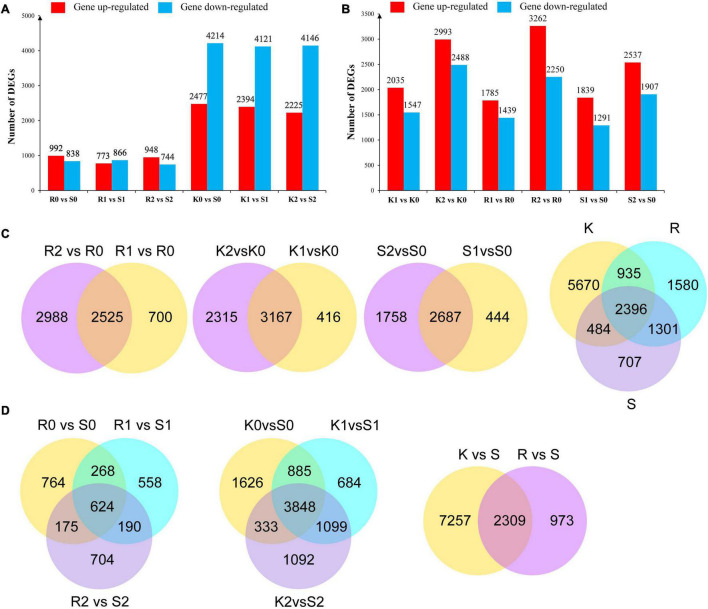
Different expressed genes in different subgroups. **(A)** Differential genes up and down regulated after inoculation *Xap* of PI 319443, HR45 and Bilu. **(B)** Comparison of differential genes between genotypes. **(C)** Venn diagrams of DEGs of K/R/S at 0, 24, and 48 h time points after *Xap* inoculation. **(D)** Venn diagram of DEGs in response to resistant genotypes. Each box represents genes being up-regulated (red) and down-regulated (blue).

To further identify genes putatively involved in the resistance response, DEGs were identified among different subgroups of R0 vs. S0, R1 vs. S1, R2 vs. S2, K0 vs. S0, K1 vs. S1, and K2 vs. S2. Compared with the susceptible genotype Bilu at the same inoculation time point, 992, 773, and 948 genes were upregulated in R at 0, 24, and 48 hpi, respectively, and 2,477, 2,394, and 2,225 genes were upregulated in K at 0, 24, and 48 hpi, respectively, while 838, 866, and 774 genes were downregulated in R at 0, 24, and 48 hpi, respectively, and 4214, 4121, and 4146 genes were downregulated in K at 0, 24, and 48 hpi, respectively (Figure 2A).

To identify the *Xap* response-related DEGs, different subgroups of K1 vs. K0, K2 vs. K0, R1 vs. R0, R2 vs. R0, S1 vs. S0, and S2 vs. S0 were analysed with regard to differential gene expression in response to *Xap* inoculation. Compared with the expression level at 0 hpi, 2,035, 1,785, and 1,839 genes were upregulated in K, R, and S, respectively, at 24 hpi, and 2993, 3,262, and 2,537 genes were upregulated in K, R, and S, respectively, at 48 hpi, while 1,547, 1,439, and 1,291 genes were downregulated in K, R, and S, respectively, at 24 hpi, and 2,448, 2,250, and 1,907 DEGs were downregulated in K, R, and S, respectively, at 48 hpi (Figure 2B). Combining the DEGs in K, R and S, a total of 9,485 DEGs were identified as being involved in the response to *Xap* infection (Figure 2C).

To identify common unigenes in different stages, the overlaps in each comparison are shown in a Venn diagram. The results showed that the number of genes at 24 hpi was significantly lower than that at 48 hpi, indicating that R, K, and S continued to respond to pathogen infection after inoculation. Compared to the susceptible genotype, the Venn diagram analysis indicated that the resistance-responsive genes were genotype specific and time specific, and a total of 2,039 genes were commonly expressed in both resistant genotypes (Figure 2D).

**FIGURE 3:**
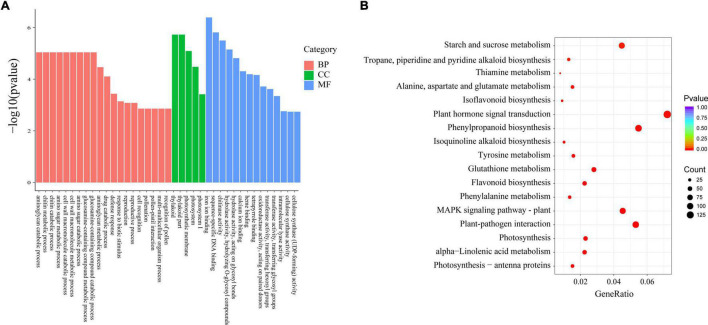
Functional analysis of 1,688 DEGs. **(A)** GO enrichment of DEGs. **(B)** KEGG enrichment of DEGs.

Considering the 9,485 *Xap* infection-responsive DEGs and 2,039 pathogen-responsive DEGs identified above, a total of 1,688 DEGs were obtained by combined DEG analysis. To further analyse the possible functions of the 1,688 DEGs, GO annotation analysis was performed to identify the major functional categories. The results showed that the 1,688 DEGs were differentially enriched in 620 terms that were dominant within the biological process, molecular function and cellular component categories, such as “defence response,” “response to biotic stimulus,” and “response to stress” (Figure 3A and Supplementary Table 6). Additionally, these DEGs were enriched in 103 KEGG pathways. Most DEGs were significantly enriched in “phenylpropanoid biosynthesis.” Some genes were significantly clustered into pathogen response-related pathways, such as “linoleic acid metabolism,” “sesquiterpenoid and triterpenoid biosynthesis,” “stilbenoid, diarylheptanoid and gingerol biosynthesis,” “flavonoid biosynthesis,” and “alpha-linolenic acid metabolism” (Figure 3B and Supplementary Table 7). These results provide a functional reference for further screening resistance-related genes.

### Identification of Co-expression Network Modules

**FIGURE 4:**
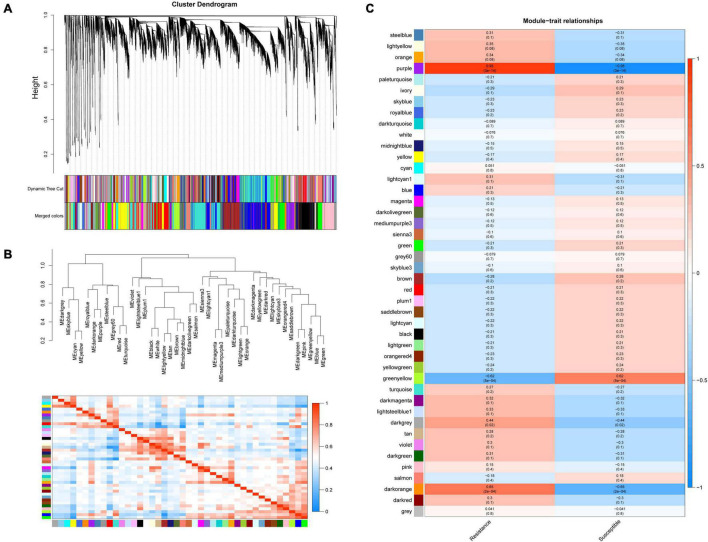
Weighted gene co-expression network analysis (WGCNA) in resistance to *Xap* infection. **(A)** Hierarchical cluster diagram of coexpression modules according to WGCNA. **(B)** Gene coexpression modules showing the cluster dendrogram (above) and the heatmap for the correlation coefficient between the modules (below). **(C)** Module–trait relationships. Each cell contains the corresponding correlation and *p* value.

After removing the genes with an average FPKM < 1, 26,053 genes were used for WGCNA to identify the candidate pivotal genes associated with the resistance to *Xap* infection. The disease resistance levels of the R, K and S genotypes were used to express their disease resistance, and the disease resistance levels of the three genotypes were correlated with 44 modules, of which three coexpression modules significantly correlated with disease resistance were obtained by screening with a *p* value < 0.01 (Figures 4A,B and Supplementary Table 8). The results showed that between the resistant genotypes, the correlation coefficient of purple was 0.95, *p* = 3e^–14^, and the correlation coefficient of darkorange was 0.65, *p* = 2e^–04^; purple and darkorange were positively correlated with resistance, indicating that these two modules may have positive regulatory effects on disease resistance, while the correlation coefficient of greenyellow was −0.62, *p* = 5e^–04^, and this module was negatively correlated with disease resistance, suggesting that one of the module genes may have a negative regulatory effect on disease resistance (Figure 4C). Of the three modules significantly correlated with resistance, 626 genes were in the purple module, 150 genes were in the darkorange module, and 544 genes were in the greenyellow module. The genes related to resistance were mainly concentrated in the greenyellow, purple, and darkorange modules (Supplementary Table 8). KEGG pathway enrichment analysis of the genes clustered into pathogen resistance-related pathways, such as “sesquiterpenoid and triterpenoid biosynthesis,” “MAPK signalling pathway,” “plant hormone signal transduction,” and “plant-pathogen interaction,” confirmed the high correlation between these modules and pathogen resistance (Supplementary Table 9).

### Comprehensive Analysis of Resistance-Related Genes

The DEG analysis and WGCNA were combined and distributed the 1,688 DEGs in 38 modules, mainly in the brown, turquoise, and blue modules. A total of 334 DEGs were associated with resistance in the three significantly related modules. Among these DEGs, leucine-rich repeat (LRR) structural domains, which are common in plant R genes, and receptor-like protein kinases, transcription factors, and oxidoreductases have also been reported to be associated with pathogen resistance, so these types of genes were the focus of this study. Eighteen resistance gene (R genes) containing LRR structural domains and nine genes containing protein kinase structural domains were identified. Nineteen DEGs belonging to the transcription factor family were also identified, encoding MYB, CPC, and bHLH transcription factors; 63 DEGs encoded oxidative factors, 37 were cytochrome P450-like DEGs, and 13 DEGs encoded peroxidase genes. Cytochrome P450 and peroxidase have been reported to have important roles in plant disease resistance.

The 139 genes annotated from the 334 DEGs were categorised into three GO categories and 74 terms. Many genes were significantly enriched in biological processes associated with defence response, response to stress, response to biotic stimulus, response to oxidative stress, response to external stimulus, signal transduction, signalling, cell communication, cellular response to stimulus, regulation of response to stimulus, and multiorganism process. Furthermore, some GO terms were significantly enriched in molecular functions among the DEGs, including haem binding, iron ion binding, oxidoreductase activity, peroxidase activity, antioxidant activity, and ADP binding (Supplementary Figure 2 and Supplementary Table 10). KEGG analysis also found that the 139 DEGs were enriched in 16 pathways. The results showed that 33 DEGs were annotated in pathogen resistance-related pathways, including “plant hormone signal transduction,” “MAPK signalling pathway,” and “plant–pathogen interaction,” and 32 DEGs were associated with the biosynthesis of secondary metabolites, such as “phenylpropanoid biosynthesis” and “isoflavonoid biosynthesis” (Supplementary Table 11).

Thirteen differentially expressed transcripts from the 139 DEGs were predicted to participate in plant hormone signal transduction, including three AP2 transcription factors (*Phvul.001G160500*, *Phvul.006G106100*, *Phvul.007G127800*), two jasmonate ZIM (JAZ) proteins (*Phvul.003G021000*, *Phvul. 003G129200*), four secretory proteins (SCRs) (*Phvul.006G196900*, *Phvul.006G197100*, *Phvul.006G197300*, *Phvul.006G197500*), two Auxin (*Phvul.001G164900*, *Phvul.002G284700*), one kinase (*Phvul.006G216400*), and one bZIP_1 transcription factor (*Phvul.009G026900*). The transcription factors AP2 and bZIP_1 are important participants in the salicylic acid (SA), jasmonic acid (JA), and abscisic acid (ABA) signalling pathways, and they exercise an important function in the phytohormone-mediated signalling pathway for disease and stress resistance as key regulators of gene expression (Supplementary Figure 3) (De Sutter et al., 2005; De Boer et al., 2011; Lu et al., 2013). The JAZ proteins TIFY6B and TIFY10A can immediately induce ubiquitin-mediated proteolysis in response to stimuli (Ebel et al., 2018). The SCR pathogenesis-related protein (PR) is involved in a variety of plant life processes in plant growth and development and in the response to pathogens (Sels et al., 2008). The above analysis results revealed the significance of plant hormones in the resistance to the *Xap* pathogen.

**FIGURE 5:**
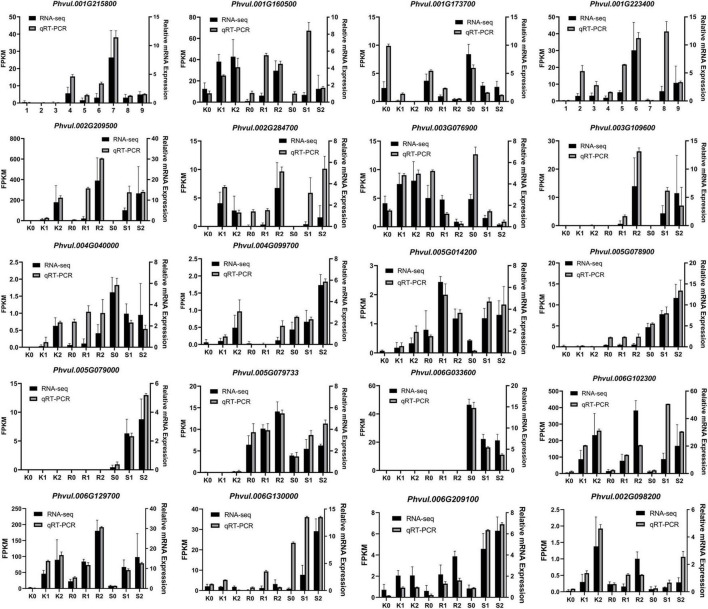
Comparison of genes expression levels using RNA-seq and qRT-PCR. K, tepary bean PI 319443; R, resistant genotype HR45; S, susceptible genotype Bilu. 0, 1 and 2 represent 0, 24 and 48 h post inoculation, respectively.

Based on the above analysis, 20 DEG expression trends in three genotypes of 139 resistance-related genes were used to verify the accuracy of the RNA-seq expression data, and RT-PCR was performed on the same RNA samples originally used for next-generation sequencing. The results showed that the 20 DEG expression trends were consistent with the RNA-seq data, demonstrating the reliability of the RNA-seq expression profile in the present study (Figure 5).

## Discussion

Common bean blight is one of the most important factors limiting common bean production worldwide. In the present study, RNA-seq was used to investigate the transcriptomic response of resistant and susceptible common bean and tepary bean genotypes during their interaction with *Xap*. More importantly, our work provided novel insights for closely related species of two resistant genotypes that were used to analyse the changes in plants at the transcriptional level when inoculated with the pathogen *Xap*, since PI 319443 is the HR45 parent line and has the same resistance loci as HR45, which has been indicated in a previous study (Singh and Miklas, 2015). The results of pathogenic bacterial colonisation by inoculation showed that PI 319443 and HR45 were highly resistant genotypes, while Bilu was a highly susceptible genotype. Due to the advantages of transcriptome analysis in the study of plant-pathogen interactions, multiple loci of resistance-related genes could be identified more easily. *Xap* has been studied by transcriptomics in rice (Zhang et al., 2015; Fan et al., 2019; Bakade et al., 2021), tomato (Shi and Panthee, 2020), pepper (Gao et al., 2021), citrus (Jalan et al., 2013; Ferrasa et al., 2020), sugarcane (Ntambo et al., 2019), and common bean (Foucher et al., 2020). Additionally, due to the advantages of WGNCA in gene function analysis, both differential expression analysis and WGNCA were used in the present study. Through in-depth analysis of the DEGs of the three genotypes, insights were gained into the candidate genes and metabolic pathways after *Xap* inoculation. Among these DEGs, genes related to resistance, peroxidase activity, plant hormone signal transduction and transcription factors were prevalent and dominant.

The plant disease resistance response is a multilevel regulatory process in which R genes play a crucial role, among which TIR-NB-LRR (TNL)-type R genes containing the TIR (Toll/interleukin-1 receptor like) structural domain have an important role. TNL-like R genes are thought to be associated with the initiation of plant defence response signals and with pathogen recognition, and in structural studies of the flax stripe rust resistance gene L6, it was found that mutating amino acids in the conserved structural domain of TIR resulted in the disappearance of plant hypersensitive responses (Bernoux et al., 2011). There were 17 R genes observed by gene annotation, among which, analysis of the identified *Phvul.002G098200* gene revealed that this gene has a TIR structural domain (Supplementary Table 12); the comparative analysis showed that this gene is homologous to the cotton *GhDSC1* gene, which responds to the JA pathway and increases reactive oxygen species levels, thereby enhancing resistance to Verticillium wilt (Li et al., 2019).

Protein kinases play crucial roles in plant resistance to pathogens. Plants recognise pathogens mainly through pattern-recognition receptors (PRRs) on the cell membrane surface, through microbe-associated molecular patterns (MAMPs), or through nucleotide-binding LRRs (NB-LRRs) as effector proteins. This process is involved in protein kinase activation, the Ca^2+^ signalling pathway, hormone biosynthesis, oxidative burst and transcriptional reorganisation through crossover or specific signalling pathways (Swiderski and Innes, 2001; Bundó and Coca, 2016). Among them, serine/threonine-protein kinase (SRK), LRR-like protein kinase (LRR-RLK) and calcium-dependent protein kinase (CDPK) have been shown to play an important role as secondary messengers. Here, one SRK (*Phvul.006G216400*) and one CDPK (*Phvul.009G082532*) were identified as upregulated DEGs, and one LRR-RLK (*Phvul.007G265100*) was identified as a downregulated DEG, in the three genotypes. KEGG analysis showed that three kinase genes were involved in “MAPK signalling pathway-plant” and “plant–pathogen interaction” pathways, indicating their important functions in *Xap* resistance.

Plant hormones play important roles in plant disease resistance. The plant defence system can activate the early infection signal induced by pathogens through the plant hormones SA, ABA, ethylene (ET), cytokinin (CTK), and gibberellin (GA) to effectively activate the plant immune response. In this study, 17 DEGs were significantly enriched in the plant hormone signal transduction pathway. JA is a key endogenous growth regulator of plant defence responses to pathogenic microorganisms (Chini et al., 2007). Two JAZ genes (*Phvul.003G021000*, *Phvul.003G129200*) and one JAR gene (*Phvul.005G106500*), acting as repressors of JA responses, were identified. Previous studies have shown that plants use JAR1 to synthesise JA-isoleucine (JA-Ile), the active form of the hormone, and subsequently recruit JAZ proteins to regulate the response of the JA gene and, in turn, the JA response. Furthermore, three AP2 transcription factors and one bZIP_1 transcription factor were found (Wu et al., 2011). The three AP2 transcription factors involved in the MAPK signalling pathway were highly induced in the response to pathogens and might protect plants against pathogen infection by regulating the JA signalling pathway.

DNA methylation is a common epigenetic modification that is closely related to gene expression regulation, transposon silencing and heterochromatin formation. The regulatory mechanism of DNA methylation can stimulate a defence response when plants are exposed to pathogenic bacterial infection (Hegde et al., 2021). Here, two caffeoyl-CoA *O*-methyltransferases (*Phvul.002G305200* and *Phvul.003G242451*) were homologous to ZmCCoAOMT2, which encodes a caffeoyl-CoAO-methyltransferase associated with the phenylpropanoid pathway and lignin production and confers resistance to both southern leaf blight and grey leaf spot (Yang et al., 2017). However, further efforts are still needed to elucidate the molecular mechanisms of DNA methylation in the plant defence response.

## Conclusion

In the present study, many candidate genes underlying the response to *Xap* in common bean and tepary bean were identified through transcriptome analysis, which might be of great value in CBB resistance breeding of common bean in the future. Most of these genes, including R genes and transcription factors, are involved in plant hormone signal transduction and the “MAPK signalling pathway–plant” and “plant–pathogen interaction” pathways, indicating that CBB resistance is a complex mechanism. More broadly, our results provide a better understanding of the complexity of the resistance to *Xap* and can guide future experimental studies and provide genetic resources and a theoretical basis for subsequent research on gene function and CBB resistance improvement in common bean.

## Data Availability Statement

The original contributions presented in the study are publicly available. This data can be found here: National Center for Biotechnology Information (NCBI) BioProject database under accession number PRJNA773264.

## Author Contributions

JW and PY planned and designed the experiments. LW contributed to the lines breeding. PY performed the experiments of resistance evaluation. PY and YC analysed the data and wrote the manuscript. JW and SW revised the manuscript. All authors contributed to the article and approved the final manuscript.

## Conflict of Interest

The authors declare that the research was conducted in the absence of any commercial or financial relationships that could be construed as a potential conflict of interest.

## Publisher’s Note

All claims expressed in this article are solely those of the authors and do not necessarily represent those of their affiliated organizations, or those of the publisher, the editors and the reviewers. Any product that may be evaluated in this article, or claim that may be made by its manufacturer, is not guaranteed or endorsed by the publisher.

## Footnotes

1
http://www.genome.jp/kegg/
