# Supplementary figures and images for: Regulatory Mechanisms of the Resistance to Common Bacterial Blight Revealed by Transcriptomic Analysis in Common Bean (Phaseolus vulgaris L.)
Source: Front Plant Sci. 2022 Jan 5;12:800535. doi: 10.3389/fpls.2021.800535 (PMC8767069; doi:10.3389/fpls.2021.800535)

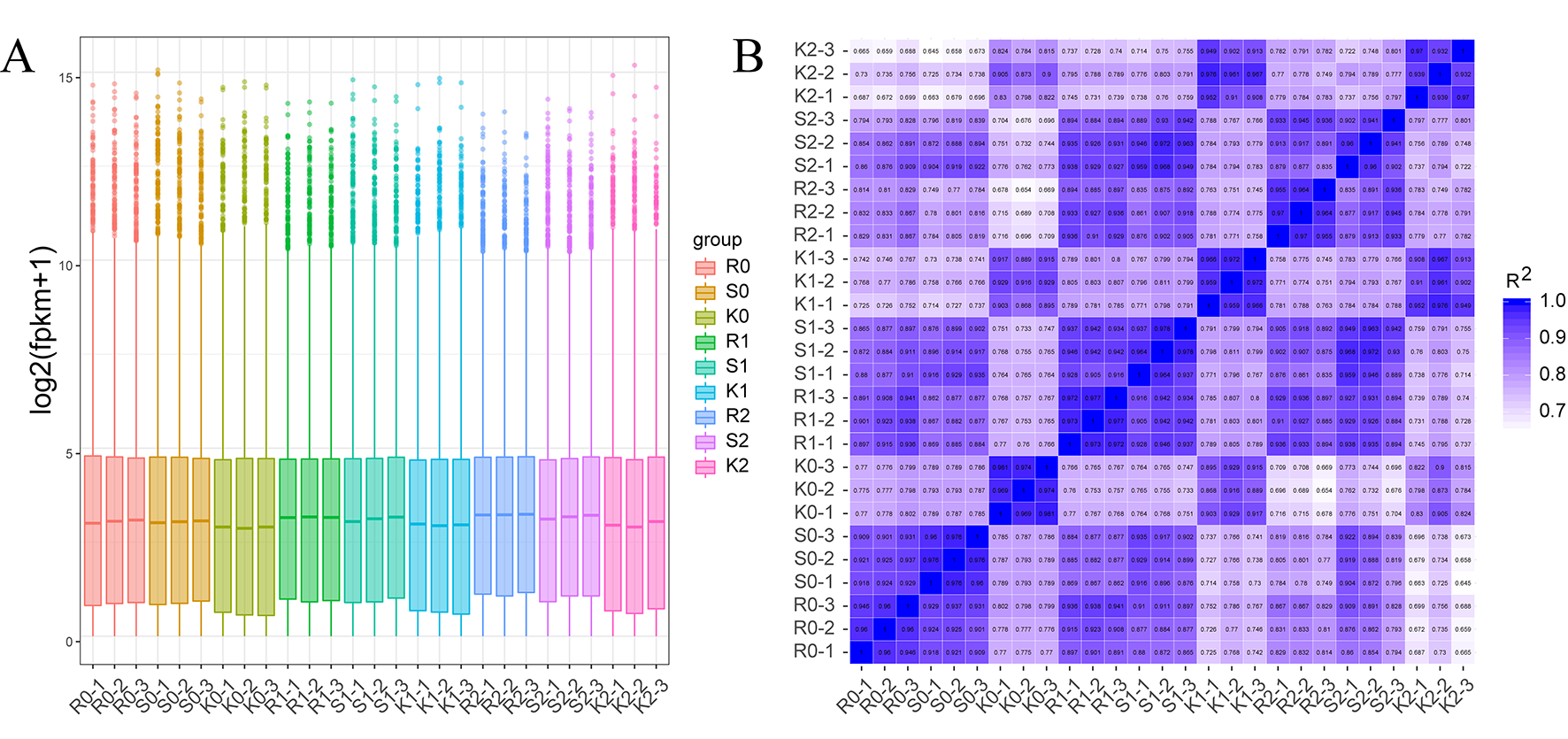

Supplement: Supplementary file 2 [file Image_1.TIF]

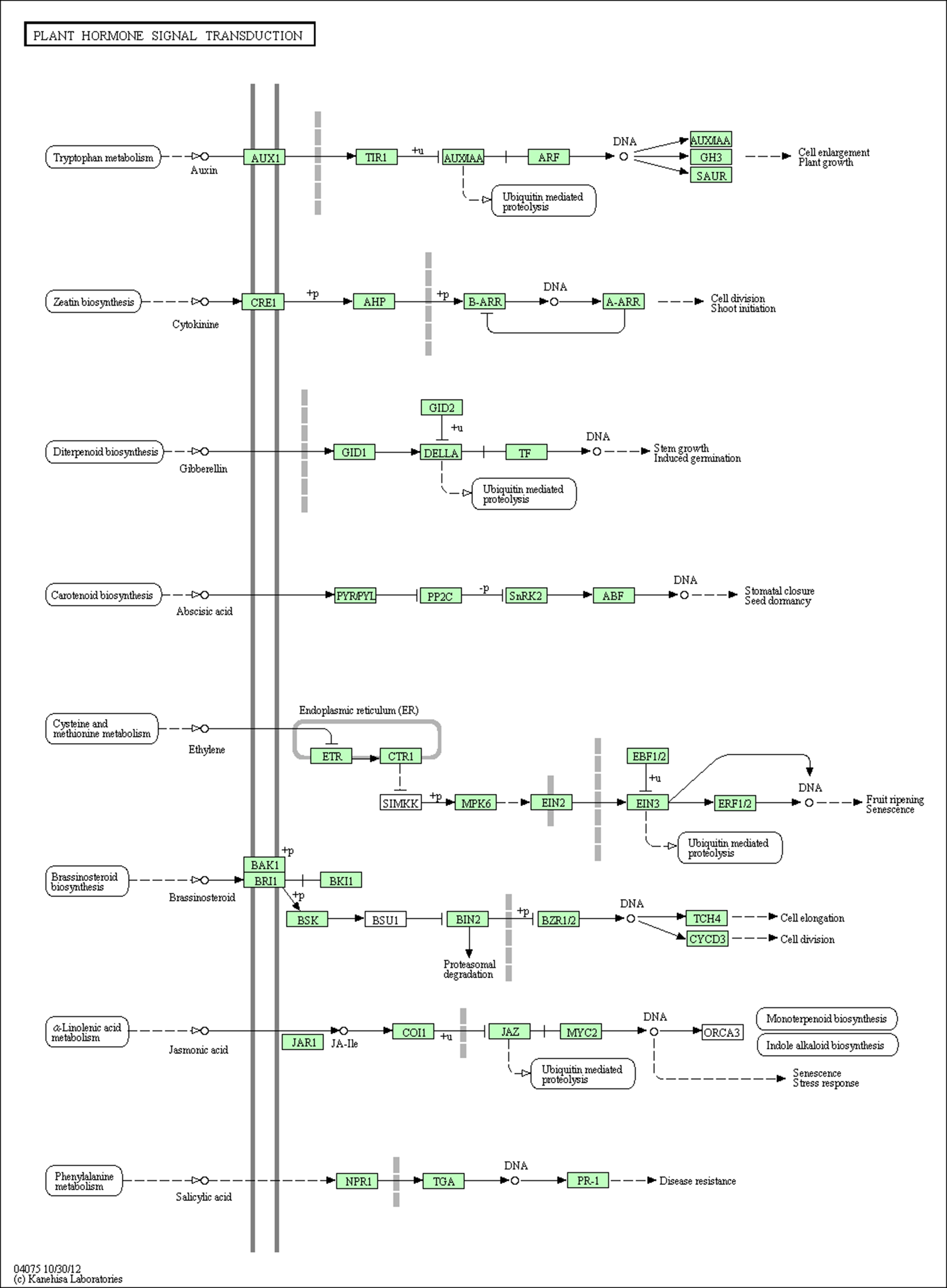

Supplement: Supplementary file 4 [file Image_3.TIF]
